# Predicting the *S. cerevisiae* Gene Expression Score by a Machine Learning Classifier

**DOI:** 10.3390/life15050723

**Published:** 2025-04-29

**Authors:** Piotr H. Pawłowski, Piotr Zielenkiewicz

**Affiliations:** 1 Institute of Biochemistry and Biophysics, Polish Academy of Sciences, 02-093 Warsaw, Poland; piotr@ibb.waw.pl; 2 Laboratory of Systems Biology, Institute of Experimental Plant Biology and Biotechnology, Faculty of Biology, University of Warsaw, 02-096 Warsaw, Poland

**Keywords:** gene expression score, *Saccharomyces cerevisiae*, random forest classifier, machine learning, AI

## Abstract

The topic of this work is gene expression and its score according to various factors analyzed globally using machine learning techniques. The expression score (ES) of genes characterizes their activity and, thus, their importance for cellular processes. This may depend on many different factors (attributes). To find the most important classifier, a machine learning classifier (random forest) was selected, trained, and optimized on the Waikato Environment for Knowledge Analysis WEKA platform, resulting in the most accurate attribute-dependent prediction of the ES of *Saccharomyces cerevisiae* genes. In this way, data from the Saccharomyces Genome Database (SGD), presenting ES values corresponding to a wide spectrum of attributes, were used, revised, classified, and balanced, and the significance of the considered attributes was evaluated. In this way, the novel random forest model indicates the most important attributes determining classes of low, moderate, and high ES. They cover both the experimental conditions and the genetic, physical, statistical, and logistic features. During validation, the obtained model could classify the instances of a primary unknown test set with a correctness of 84.1%.

## 1. Introduction

Gene expression is a manifestation of a gene’s role in a cell, usually through the processes of RNA transcription and protein synthesis [1]. It is, therefore, a manifestation of the “movement” of the biochemical machinery in the process of life. Gene expression may be divided into the following stages: signal transduction, chromatin remodeling, transcription, posttranscriptional modification, RNA transport, translation, and mRNA degradation. There are many views on what controls protein expression, which can be studied [2]. At the level of molecular biology, three determinants of this phenomenon are widely recognized [3], i.e., transcriptional regulation, e.g., by transcription factors and structural DNA properties [4]; the modulation of the transcription machinery, e.g., by accessory factors [5] and ligands [6]; and epigenetic structural influence, e.g., involving the chromatin remodeling process [7]. All of these are focal points of interest in cell biology and have recently been intensively investigated, mainly because of the development of large repositories that collect exabytes of gene expression data.

The measurement of the activity of the expression of thousands of genes at once (gene expression profiling) to create a global picture of cellular function or to show how the cells react to a particular treatment is possible with the technology of DNA microarrays [8] or RNA-Seq [9].

Gene expression at the cellular level may be quantitatively characterized by the abundance or the so-called expression score (ES). The first one, i.e., the number of copies of a protein molecule in a cell, is an absolute physical quantity. The second one is a transformed relative measure. It is calculated as a log2 ratio, where the ratio represents the normalized detection amount, i.e., the quantity divided by the control value, or by the average value across all conditions [10].

Abundance may be a useful parameter in the biophysical modeling of biological processes [11], and the expression score may be a key parameter in bioinformatics prediction, classification, and comparative analysis [12]. In this work, the second approach was chosen as more universal in relation to various conditions.

Owing to the relatively high cost of gene expression profiling, the parameters mentioned above have been predicted theoretically, considering the kinetics of ribosome action [13], the Bayesian network of mRNA-related features [14], and the correlation between landmark and target genes [15].

There are many views on what governs protein expression, some of which were presented at the beginning of the introduction. Our work is a proposal for an original look at this topic and indicates the most important factors with the participation of machine learning (ML). To achieve this goal, we attempted to predict the ES of *S. cerevisiae* cells on the Weka platform with the hope of revealing important factors associated with this phenomenon. Therefore, after several attempts, the most effective classifier in the search was selected: the random forest.

A set of exemplary genetic, physical, statistical, and logistic factors were taken into account in the evaluation. In short, to achieve our task, which is a type of data mining, we looked for a suitable classification technique for analyzing the impact of assumed attributes on the known final result. In the preprocessing stage, with a set of different evaluators that evaluated the usefulness of attributes in the classification process, e.g., by measuring the Pearson correlation coefficient or the information gain coefficient with respect to the class of the expression result, we narrowed the initial set of 472 potential attributes to 19 relevant attributes.

After preliminary trials with different classifiers, the random forest classifier was selected as the main tool to perform the analysis. The trees of this forest are networks of nodes and edges that describe decision-making processes.

During the training process, the random forest algorithm analyzes each decision tree characterized by random sets of attributes assigned to nodes and decision rules assigned to edges from the point of view of their impact on the final classification decisions for the entire training set. It can handle both nominal and numerical data and fits a set of decision trees that are the best in a given classification task.

With the help of this tool, we created an optimal random forest for the classification of the expression score size and identifying the best decision paths, indicating the most important cases in a set of important attributes. In our project, we abstracted from a single gene and specific conditions and analyzed the global picture of the dependence of the gene expression score on many coexisting factors. Finally, we attempted to reveal the main rules or chains of relations that determine the intensity of expression. In this way, we developed a classification model that connects expression scores with basic features of different natures.

## 2. Materials and Methods

### 2.1. Introduction to ML: Basic Concepts

One of the most important advantages of ML [16] is the possible improvement in the performance of some sets of complex algorithmic tasks. This is why such an approach has been implemented recently in difficult bioinformatics areas [17]. The improvement comes mainly from the technique of model training.

**Figure 1:**
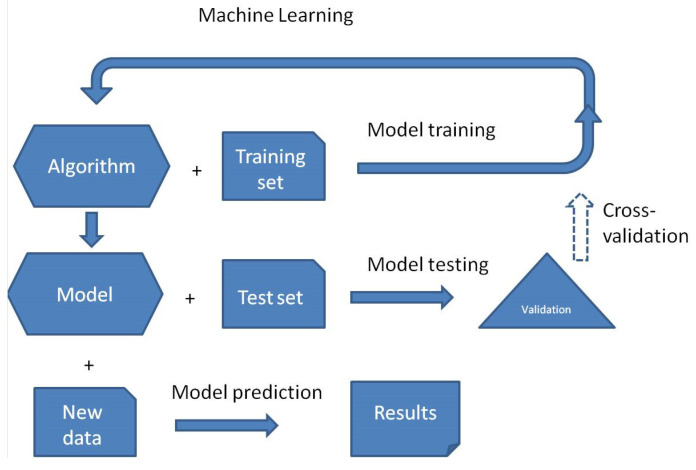
Main stages of machine learning. Cross-validation is an option.

ML model training is the process of teaching an algorithm to find specific patterns and then predict proper outcomes, carried out by exposing it to labeled data. This approach starts with random parameters that are repeatedly modified to minimize the discrepancy between its predictions and the training data labels and ends with the validation test of the final model and then possible predictions (Figure 1). The validation of the final model may be performed by using the test set of unseen data and/or by k-fold cross-validation, in which the training set is randomly partitioned into k groups (roughly equal in size), and then, the algorithm predicts one group each time by training other (k-1) groups. The final prediction performance is the average of k repeated predictions.

**Figure 2:**
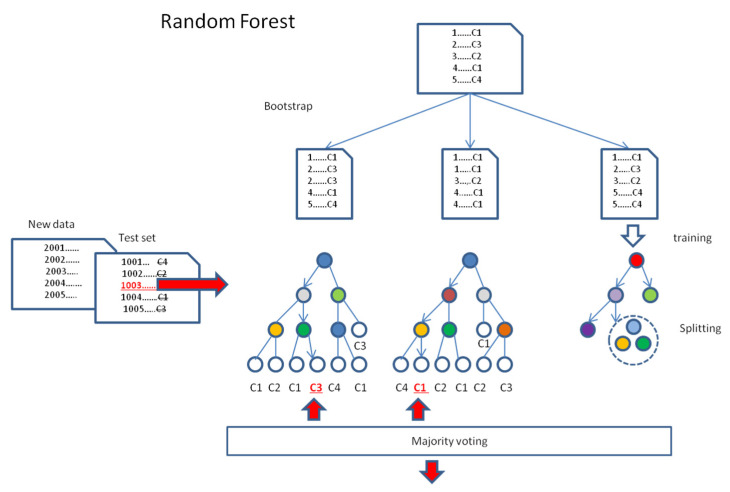
Random forest model. In this algorithm, the training dataset is bootstrapped into equal random samples with replacement. Next, each data sample is used for training the separate tree, which grows in the process of splitting. Trained trees may be tested or used for the prediction of the class of new data. The final classification decision is always made by majority voting of all the trees. Red color indicates the item of interest, and underlining indicates the particular decision.

A random forest [18] is a machine learning model that combines multiple decision trees to predict the data item class in the classification task (Figure 2). Initiating at the starting node and moving the data items along the tree branches, at each node passed, a decision is made as to which branch of the tree they should proceed. In practice, it depends on an algorithmically specified node attribute and its split point value (see below), according to which the items are sorted. Finally, the items are assigned to the terminal nodes of the tree, called leaves. If many items labeled by class are sorted by tree (training) in this manner, the number of representatives of each class in a given leaf can be determined, and thus, the classes dominating the leaves can be determined. If an item with a formally nonlabelled or unknown class is tested or predicted by the tree, the dominating class of the leaf node, which is terminal for the item, may be taken into account as a vote when further classifying. Therefore, for classification tasks, the forest performs the prediction by majority voting among all the trained trees, examining the same item. In such an approach, the “wisdom of crowds” is used, in which the collective decision-making process has the property of averaging errors and offering more reliable predictions. This is usually its advantage relative to individual trees.

During the training of the forest of random decision trees, which is an independent process for each tree, the classifier algorithm chooses (the implementation of the method known as bagging or bootstrap aggregation) the individual random subset of equal-size training data (with replacement) for classification by the tree. Next, a given tree is constructed, aiming at the greatest degree of differentiation of the split data. It is performed on the basis of the analyzed data attributes, which are taken as nodes, and the best-split point values are implemented in the adequate decision rules, which are assigned for the driving edges (branches). During the process of splitting, at every stage of tree growth, the child nodes are selected from the random subsets of possible attributes to guarantee further splitting of the processed data into the subsequent subsets, resulting in a maximal decrease in the mean impurity of classes, represented by their elements. The tree local growth continues until the pure node, or the predefined final conditions are reached (e.g., maximum depth of the random forest, i.e., the longest path from the root node to the leaf node). Finally, all the leaf nodes end with a single classification vote, indicating the majority class.

The majority voting of the forest of trained trees is used in classifying the instances of the test or the new data. It can handle both categorical and numerical data and offers the best set of decision trees for a given classification task.

### 2.2. Main Stages of the Applied Procedure

Today, ML technology operates with a large amount of data. It often requires time- and cost-consuming preprocessing to ensure the data’s proper quality, uniqueness, relevance, context, and balance and to avoid hidden incomprehensible complexity. To minimize the above task for the prediction of ES, we first decided to consider genes from only 4/16 chromosomes, especially the chosen chromosomes, which represent the diverse content of genome regions with slow, moderate, and fast replication speeds [19]. Additionally, the range of analyzed nucleotides and amino acid sequences for a given gene was specifically limited to the vicinity of the start codon, covering several tens to hundreds of items, both on the coding and noncoding sides. Genome segments of this size are long enough to form RNA stem loops [20] and locally folded protein globular domains [21].

After initial minimalization, but still in the preprocessing stage, we evaluated the worth of the potential attributes for the classification task. A set of six different kinds of attribute evaluators from the Waikato Environment for Knowledge Analysis (WEKA) were used. For example, CorrelationAttributeEval evaluates the value of an attribute by measuring the correlation (standardized covariance) between it and the class, whereas GainRatioAttributeEval evaluates the value of an attribute by measuring the information gain ratio concerning the class. By comparing the average values of differently estimated worth, we narrowed the set of the 472 attributes to the 19 most essential variables.

To achieve our main task, which is a type of data mining and can benefit from the application of ML, we tried to choose the best classification technique for analyzing the impact of the most essential attributes on the expression score. After preliminary trials with different optional classifiers from WEKA, trained, cross-validated, and independently tested, a random forest classifier was selected. Its optional hyperparameters were additionally tuned by an empirical process of trial and error, finally defining the optimal tool for preparing this study. Using the random forest method, we constructed an effective classifier for the expression score and marked the best decision routes, with the most important elements in the set of essential attributes indicated.

### 2.3. The Data Platform for AI

WEKA, Waikato Environment for knowledge analysis version 3.8.5, was applied [22].

### 2.4. Initial Data

**Figure 3:**
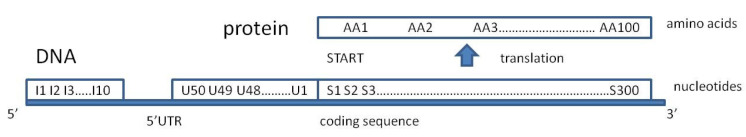
Biological mining of genetic type attributes representing nucleotides (Ui and Si) along the DNA strand and 3-letter nucleotide codons translated into amino acids (AAi) in the process of protein synthesis, according to the universal genetic code. Always start codon S1S2S3 = ATG and amino acid AA1 = M (methionine).

The data (998,000 records) describe the expression score and the 472 prechosen attributes for *S. cerevisiae* genes located on chromosomes I, VI, XI, and XVI. They include the results of wild-type yeast heat shock (WTH) experiments [23], ACY142 strain glucose introduction (Glu) experiments [24], and diamide addition (Dia) to culture experiments [25], which were taken from the SGD database [26]. In WTH experiments, strains were grown at 25 °C and shifted to 37 °C. Samples were taken 0, 5, 15, and 30 min after the shift. In Glu experiments, cells were exposed to 2% glucose for 20, 90, or 150 min. In Dia experiments, 1.5 mM diamide (Sigma, St. Louis, MO, USA) was added to the culture, and samples were recovered at 5, 10, 20, 30, 40, 60, and 90 min. The attributes were chosen according to personal experience and the previous knowledge of the authors to cover many levels of cellular organization and their relationships with the environment and experiments. The nominal attributes were as follows: Exp{WTH, Glu, Dia} is the experiment type; Chro{I, VI, XI, XVI} is the order number of chromosomes; Fun{1…358} is the function assigned on the basis of the recommended three-letter gene name; Ii{A, T, G, C} is the first ten 5’ terminal bases of the 5’UTR (->3’ direction) (Figure 3); Ui{A, T, G, C} is the bases of the 5’UTR in the fifty subsequent positions directly adjacent to the start codon (->5’ direction); Si{A, T, G, C} is the subsequent bases at the first three hundred positions of the coding sequence; and AAi{A, C, D, E, F, G, H, I, K, L, M, N, P, Q, R, S, T, V, W} is the first one hundred subsequently coded amino acids, according to the universal genetic code [27]. The numeric attributes were as follows: 5’UTRL, the length of the 5’ untranslated region; TransL, the length of the transcript; ProtL, the length of the protein-coding sequence; MM, the molecular mass of the protein (Da); nA, nT, nG, and nC, the number of adenine, thymine, guanine, and cytosine residues, respectively, in the analyzed coding sequence (per 300 nucleotides); and time{5, 10, 15, 20, 30, 40, 50, 60, 90, 150}, the time of the experiment (minutes). The class attribute ES expression score from the Gene Expression Omnibus [28] was normalized and log2 transformed, a measure of the corresponding protein detection. For example, the attribute that considers chromosome number was evenly chosen such that both small (I, VI) and large (XI and XVI) chromosomes were selected to characterize the possible dependence of the expression score on chromosome size.

**Figure 4:**
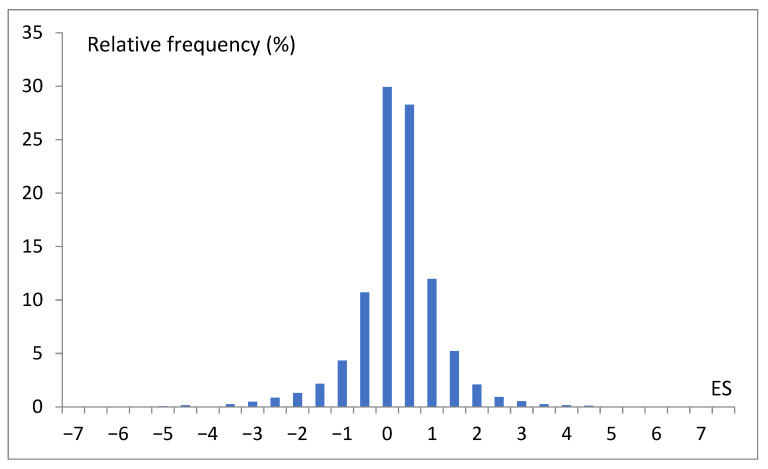
Histogram of preselected 5793 expression score (ES) records, where 0 means the range −0.5 < ES ≤ 0, and 1 means the range 0.5 < ES ≤ 1.

All instances were revised against repeatability and incompleteness. As a result, 5793 unique records were preselected, and a histogram of cases was prepared (Figure 4). These records were randomly divided concerning genes into a training set (5593) and a test set (200). The last one refers only to the genes absent from the training set.

### 2.5. Data Balancing

The attribute ES, being a real value feature, was replaced by the nominal class attribute LMH{L, M, H}, according to the following relations:

LMH = L for ES < −1(1)

LMH = M for −1 ≤ ES ≤ 1(2)

LMH = H for ES > 1(3)

Then, both the training set (5593 records) and the test set (200 records) were randomly balanced to cover the same number of records of a given class, resulting in 3 × 523 and 3 × 21 records, respectively. Balanced sets were assumed in all the following considerations.

### 2.6. Attribute Selection

**Figure 5:**
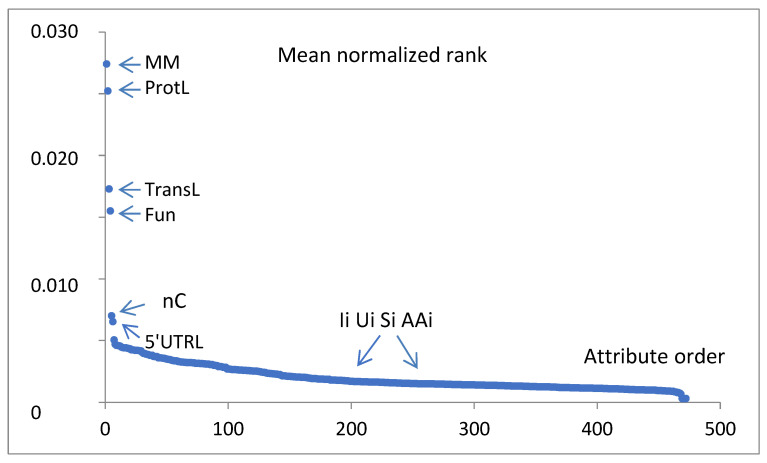
Six WEKA attribute evaluators GainRatioAttributeEval, CorrelationAttributeEval, OneRAttributeEval, InfoGainAttributeEval, ReliefFAttributeEval, and SymmetricalUncertAttributeEval were applied to the training set, and the Ranker search method was chosen. The obtained ranks were normalized and averaged in the set of considered evaluators. The values vs. attribute ranking order are presented. The leading attributes are indicated by short names.

To find the most significant attributes, which best indicate the gene expression classes of the analyzed records, six WEKA attribute evaluators (weka.attributeSelection), i.e., GainRatioAttributeEval, CorrelationAttributeEval, OneRAttributeEval, InfoGainAttributeEval, ReliefFAttributeEval and SymmetricalUncertAttributeEval, were applied to the training set, and the dedicated Ranker search method (weka.attributeSelection. Ranker -T -1.7976931348623157E308 -N -1) was chosen. The obtained ranks were normalized (divided by the total sum for a given evaluator) and averaged in the set of considered evaluators. The full mean normalized attribute ranking is presented in Figure 5. Only a few ranks of leading attributes dominate over the rank of 0.005 (the exact values are presented in Table 1). Many more attributes are poorly distinguished. The top-ranked mean normalized attributes are of nongenetic meaning, i.e., MM, ProtL, TransL, Fun, nC, and 5’UTRL.

The mean normalized ranks for the characteristic groups of attributes are 0.019—physical properties, 0.009—logistic, 0.005—statistical, 0.003—experimental conditions, and 0.002—genetic.

Owing to the above numeric evaluator findings indicating the overall domination of the nongenetic attributes, in the following analysis, all the leading nongenetic attributes were applied. On the other hand, classification methods may combine many specific decision rules, and then, the context may amplify the strength of the other parameters. To enable this additional possibility, we also represented the dominant majority of the other attributes. The arbitrarily added features indicate the experiment type, the chromosome number, the number of nucleotides of the respective type, and the chosen genetic attributes of the hypothetical possible meaning in the neighborhood of the start codon (coding and noncoding positions). The Ii attributes, containing the smallest group (10) of nucleotide sequence-related attributes, describing the sequence positions relatively far from the start codon, were first omitted. The final list of analyzed instance attributes (19) and the class attribute is presented in Table 2 and is divided into selected aspects of features and conditions. The presented attribute order was arbitrarily chosen and conserved in all machine learning tasks.

## 3. Results

### 3.1. Finding the Optimal Model

To find the optimal LMH classification model, first, the ten arbitrarily chosen WEKA classifiers were trained on the training set, and then, it was tested on the testing set (Table 3). In machine learning, the considered attributes were approved according to the selection shown in Table 2, and in the fitting, the optional WEKA parameters were applied. The best attempt was performed via random forest, with one hundred trees (correctly classified tested instances cci = 77.8%, and 10-fold cross-validation (CV_10_) for the training set cci = 83.9%).

To check whether the addition of the other attributes may improve the correctness of classification with the random forest algorithm, the training and test probes were repeated twice: the first time, with the 10Ii attributes added (test cci = 63.5%), and the second time, with all 472 prechosen attributes included (test cci = 41.3%). The resulting lower correctness (cci) indicated that in the further stages of the research, only the previous 19 selected attributes should be applied.

Then, the random forest algorithm with the selected nineteen attributes (Table 2) was optimized in dozens of arbitrary trials to modify the optional hyperparameters of the WEKA algorithm to improve the classification results. Since these trials showed the predominant importance of two parameters, the procedure was shortened, and a systematic step-by-step search was performed in the range of 1 < seed < 20 and 0 ≤ MaxDepth ≤ 20. Finally, the key parameters seed = 8 and MaxDepth = 9 were set, and the following optimized scheme was implemented:

weka.classifiers.misc. InputMappedClassifier -I -trim -W weka.classifiers.trees. RandomForest -- = -P 100 -print -attribute-importance -I 100 -num-slots 1 -K 0 -M 1.0 -V 0.001 -S 8 -depth 9

The above modification increased the number of correctly classified instances of the test set, up to cci = 84.1% (the training set CV_10_ cci = 83.5%). The total number of terminal leaves in the resulting optimal forest is 204,630. This, on average, yields 0.77 assigned instances per leaf in the forest. In the training process, only 22% of the possible leaves were used. In the training set, the mean percentage of true positive predictions per applied leaf was 99.86%.

The analysis of optimal model attribute importance was based on the decrease in average impurity when a given attribute was included and is presented in Table 4, with the number of nodes using that attribute. These results were estimated for a full training set (cci = 100%).

**Figure 6:**
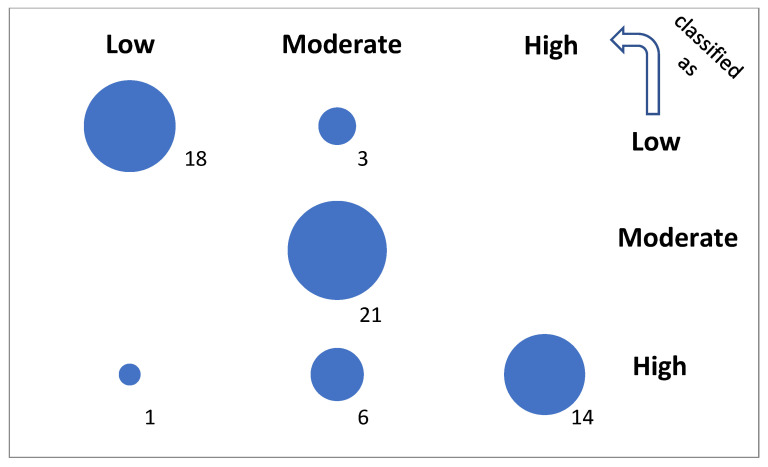
Confusion of the optimal model. The diagonal represents true predictions. The off-diagonal represents poor and very poor predictions at small and greater distances from the diagonal, respectively.

A confusion matrix for the final classification test is visualized in Figure 6. The detailed rates (TPR, FPR, TNR, and FNR) of performance and the total accuracy (ACC) by class are presented in Table 5. They were calculated with the basic performance metrics, i.e., the number of true positive (TP), false positive (FP), true negative (TN), and false negative (FN) classified samples, as follows:

TPR = TP/(TP + FN)(4)

FPR = FP/(FP + TN)(5)

TNR = TN/(TN + FP)(6)

FNR = FN/(FN + TP)(7)

ACC = (TP + TN)/(TP + TN + FP + FN)(8)

**Figure 7:**
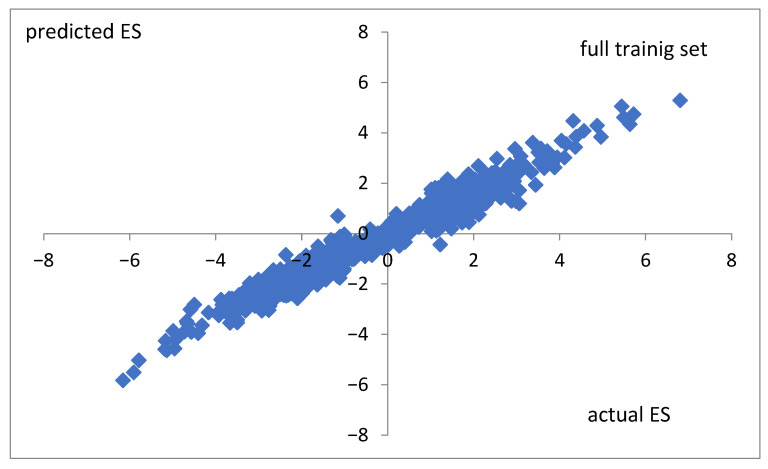
Actual values versus those predicted by the random forest classifier for non-classified ES. Data for the full training set were taken. The correlation coefficient is cc = 0.98.

To check the correctness of the predictability of the random forest algorithm in the case of real values of the expression score, the same optimized model was applied to the non-nominally, preclassified real-values ES. The correlation coefficients are cc = 0.98, cc = 0.73, and cc = 0.48 for the full training set (Figure 7), the CV_10_ training set, and the test set, respectively.

### 3.2. Example Topology of the Tree

**Figure 8:**
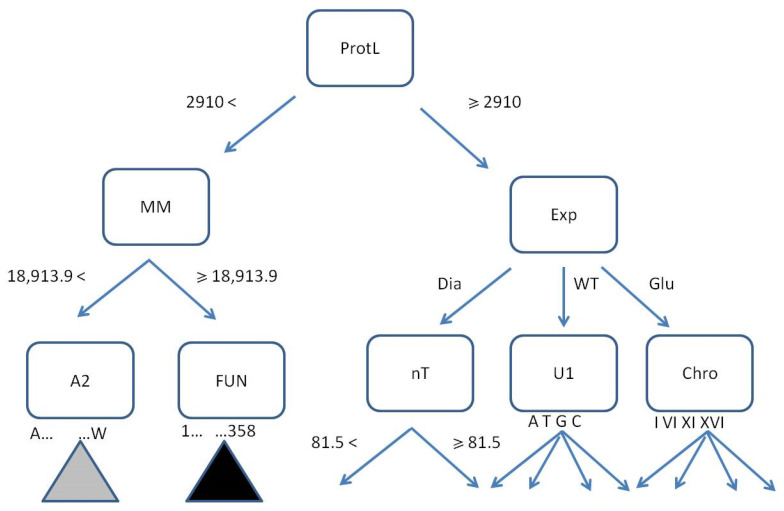
The first tree in the optimal random forest (only 2 levels from 9 are shown). The decision rules are ProtLength < or ≥2910, MM < or ≥18,913.9 Da, Exp = {Dia, WTH, Glu}, nT < or ≥81.5, U1 = {A, T, G, C}, Chro = {I, VI, XI, XVI}, AA2 = {A, C, D, E, F, G, H, I, K, L, M, N, P, Q, R, S, T, V, W}, Fun = {1, …, 358}. The gray and black triangles represent possible amino acids and functional routes, respectively. The classification goal requires up to 9 levels.

The initial two levels of the first tree (size 1505, max depth 9) in the optimal random forest are presented in Figure 8.

### 3.3. Best-Predicting Routes

The analysis of the training result buffer led to indication routes for the best prediction of a given class (Table 6) in terms of counts per 1569 training records.

### 3.4. Finding the Dependence of the Test Classification Correctness on the Presence of the Selected Attribute

**Figure 9:**
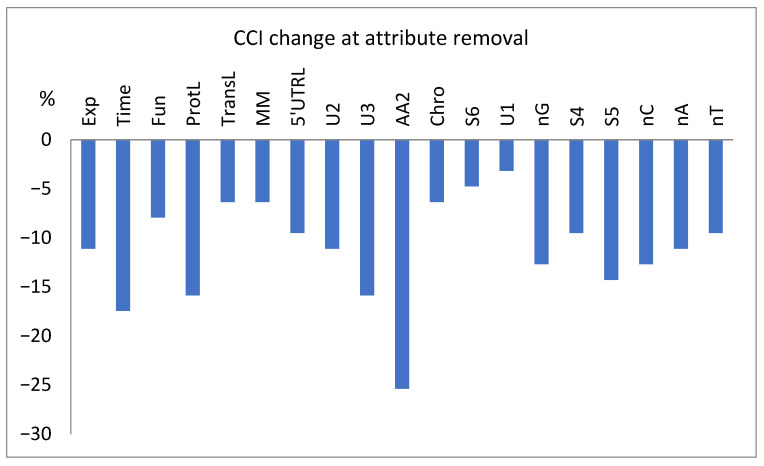
Percentage change in the number of correctly classified tested instances (cci) for the classification attempts with the exclusion of selected attributes.

The dependence of the test set classification results on the presence of a given attribute is presented in Figure 9, where the decrease in the number of correctly classified instances (cci) is shown for the attempts with selected attributes excluded from the model.

## 4. Discussion

The general goal of our work was to abstract from a single gene a holistic review and an assessment of the widest possible spectrum of factors that can affect protein expression, starting with the informational and physical specificity of DNA, whether it is transcribed or not, and ending with the conditions controlled in the experiment. The scope of our studies also included nucleotide statistics, DNA localization, and the primary structure of proteins and their functions in the cell. The real objectives of our work were to identify the attributes and leading systems that determine exemplary protein expression in *S. cerevisiae*, a species that is very popular in laboratory practice. In this work, the analyzed gene expression profile was real, and the expression score was chosen as a more universal characteristic than the abundance, especially concerning various conditions. Our proposal of an original look at this topic and a comprehensive indication of the most important factors was supported by the innovative use of artificial intelligence (AI) to perform complex biological, chemical, and physical analyses.

Gene expression, which is essential for the functioning of a cell, is usually analyzed by the statistical summarization of sample data and is often presented in histograms or expression profiles [37]. The expression score is understood as the log2 of the ratio of the protein level of a given gene to the reference level and is usually the determinant of the change in gene activity according to the current specific cell function or the response to environmental stimuli. In this work, the reference level for dual-channel data was typically loaded as the control; whereas, data from single-channel arrays were normalized with the average expression for each gene across all conditions [10]. Similar solutions have been presented in the literature [38,39,40].

Different data are collectively presented in Figure 4. To indicate the overall dominance of the *S. cerevisiae* genes with moderate expression scores (range: 0.5–2 × mean value). This picture also suggests that, at first approximation, we may consider three levels of gene expression, i.e., low (L), moderate (M), and high (H) expression scores. In our project, we abstracted from a single gene and specific conditions and analyzed the global picture of the dependency of the gene expression score on many coexisting factors. Finally, we attempted to reveal the main rules or chains of relations determining the expression intensity. In this way, we developed a classification model that relates expression scores with the basic features of different natures. In our research performed with WEKA, which is a very convenient environment for fast data mining, each ES was turned into the trivalent nominal attribute low–moderate–high (LMH).

In the days before the era of machine learning, the analyzed attributes of the objects being studied were selected by the investigators in the best possible accordance with the current state of knowledge. In the above analysis, we decided to include the advisory help of algorithms in this matter. The initial trials of estimation of the utility of attributes for a planned task with the minimized training set (1569 instances and 472 attributes) based on six WEKA attribute evaluators (i.e., GainRatioAttributeEval, CorrelationAttributeEval, OneRAttributeEval, InfoGainAttributeEval, ReliefFAttributeEval, and SymmetricalUncertAttributeEval) showed the importance of nongenetic features (Table 1, Figure 5) dominating over a rank of 0.005, and the other attributes were poorly distinguished. Among them, the least important group was the group of genetic attributes (mean normalized rank 0.002). As there was no a priori threshold value of rank considered, we decided to use all 12 nonsemantic (physical, logistic, statistical, and experimental conditions) variables and only 7 of 460 prechosen genetics (Ii, Ui, AAi, and Si) from potentially important positions of DNA and protein strain (U3, U2, U1, S4, S5, S6, and AA2). Thus, the proposed final list of analyzed attributes (Table 2) contains only 19 items. This result complemented the initial minimization and was not challenged by subsequent attempts to extend the attributes set. Good balancing of the data to cover all considered classes equally resulted in 1/3 of the instances being correctly classified by the ZeroR majority classifier (Table 3). Further investigation of the best classifier for LMH resulted in the selection of a random forest classifier (cci = 77.8%).

The two probes of the extended random forest model, trained and tested with the addition of the attributes describing the initial sequence (Ii) or all of the prechosen attributes, yielded test results of cci = 63.5% and cci = 41.3%, respectively. The small importance of the initial sequence and other parts of the genes may be surprising. This finding indicates that, in general, the sequences closer to the start codon for translation, both before and after translation, are more important than others are. The others may be the main source of the classification errors in the proposed method.

The not extended but optimized version of the model, with only 22% of possible leaves assigned (204,630), yields cci = 84.1% in the testing set containing nontrained genes. A high accuracy result for a relatively small test sample may be due to sampling error or overfitting, but the similar CV_10_ result obtained for larger samples shows that this is not the case. This optimal model favors experimental conditions (Exp and Time) on the attribute importance list (Table 4) during the training process. Next are the gene function (Fun) and the physical parameters (ProtL, TransL, MM, and 5’UTRL) of the protein and transcript, which confirms the initial findings of significant attributes independent of the classification method (Figure 5, Table 1), except for the statistical attribute nC. The final result is 16% worse than that for the full training set (100%). Here, the number of true positives is equal to 18, 21, and 14 for the L, M, and H classes, respectively, per 63 tested instances (Figure 6, Table 5). A total accuracy (ACC) greater than 0.85 is a good result.

The same random forest model applied to non-nominally, preclassified real values of the expression score (ES) yields correlation coefficients of cc = 0.98, cc = 0.73, and cc = 0.48 for the full training set, the CV10 training set, and the test set, respectively. This result shows that even though a more precise method (real values) offers a good explanatory description of the known results (Figure 7), it may produce a less correct forecast of unknown changes in the expression score (test set). Such a case suggests possible overfitting (trained fitting to errors) or a distributional shift in the test data. It seems that nominal classification (L, M, H) proposed in this paper may minimize these unwanted effects.

An example of a single tree branch shows the root node ProtL with the net of attributes, both binary and multidirectional, splitting the instances according to the rules represented by edges (Figure 8). Terminal leaves indicating assigned classes, usually present at deeper levels, may be filled with drawn and segregated instances or not. If yes, they become potentially predictive.

The best-predicting routes in the forest contain up to 6 levels (Table 6) and may repeat the same attributes with differently defined rules (constraints). Here, the most often used attribute is ProtL. The results show that there are 71 instances in which highly expressed genes produce proteins that are not shorter than ProtL = 469.5 and lighter than MM = 71,288.1. They have a 5’UTRL region that is not shorter than 72 and obeys the genetic rules S5 = T, U3 = A. These effects are especially manifested in experiments with 1.5 mM diamide. The presence of adenine at the -3 position is consistent with the fact that it most often occurs in the Kozak sequence [41]. Thymine is reported at positions +4 and +6, giving way to cytosine at position +2. Our results may be specific to diamide-treated cells.

As shown in Figure 9, the estimated importance of the attributes during the selection (Table 1) and training processes (Table 4) does not translate directly to importance during the test classification process. Ignoring some genetic attributes (e.g., AA2, U3, and S5) can seriously decrease the correctness of the final test classification (Figure 9). This may be related to the absence of test set data in the training, overfitting, or possibly accidental specific importance of the mentioned genetic attributes in these sets of data, i.e., amplified by the context (see below). In general, it clearly shows that less numeric importance is not equivalent to absence, which may prevent the correct analysis of many complex dependencies, e.g., due to the threshold effects. Thus, despite the genetic attributes that might not be the most important, they cannot be neglected without consequences.

The process of model training also reveals the greater importance of the experimental conditions, i.e., Exp and Time (Table 4), than the functional, physical, logistic, genetic, and statistical attributes revealed by attribute evaluators, e.g., MM and ProtL (Table 1), which may be related to contextual dependencies or feature interaction effects not taken into account by evaluators. This is not confirmed in Figure 9, probably because of the small size of the test file.

A single decision route of the trained random forest model, from the root to the terminal leaf, leads through instances, obeys all the attribute constraints along the route, and assigns them a certain arbitrary given class. For the well-trained model, the ratio of the number of instances classified in this way into the proper class to the number of all classification attempts should be close to one. This ratio approximates the conditional probability of the event in which the instance meeting the conjunction of all route constraints represents a gene belonging to an algorithmically defined class. It is reciprocal to the probability of the conjunction, with other parameters remaining constant. The probability of a specific conjunction may be very small; in this way, the random forest model can use the information of very specific attribute conjunctions to determine the proper class with a very high probability of success. As such, it may be very predictive but not too universal. The best predictions include samples of 41, 120, and 71 items, for a total of 523 items per class. It also does not reveal the individual impact of a single feature. This is why a random forest classifier in practice is treated as a “black box”.

The expression score attempt, which considers descriptive attributes, differs from the abundance attempt, which analyzes the translation process from the point of view of molecular physics [13]. Thus, from a broader perspective, the discussed optimal model is a natural complement to a physical model. From this perspective, in general, the primary structure of DNA determines gene expression to a lesser extent than the experimental conditions, gene function, and the basic physical characteristics of proteins and transcripts. In particular, protein length is a very useful parameter in the most effective prediction routes. At first, the claim that the DNA primary structure has a weaker effect on gene expression than the growth conditions may seem to be contrary to common knowledge [42,43]. However, it should be remembered that it only refers to the change in expression (k-fold change), and this may depend on external factors. The authors believe that AI discovers the natural intelligence of life, which means that cells are not simple automata playing the program stored on DNA but can respond to the environmental influence adequately to the strength of the stimulus and their own capabilities and needs.

In accordance with the above, the multilevel depth of the random forest, which predicts the expression score, indicates the stronger role of the ensemble of factors (attributed along the route) than single attributes. Moreover, the attributes indicated as the most important by the ML classifiers differ from those indicated by the overall evaluators. These findings may suggest the role of a specific amplifying context (set of conditions) defined by an ensemble. This context should be analyzed in particular in future investigations. Of course, single decision-making rules are also worthy of further analysis, because they may hide the possible “bottlenecks” of the decision routes and, thus, the elementary constraints of gene expression. Therefore, the possible useful application of the random forest gene expression classifier may obey in silico experiments predicting the result of the modification of selected attributes and considering the context.

The biological rationale for the special selection of chromosomes and gene regions in this work may raise some doubts. However, we expect that the time and cost savings in the number of chromosomes considered (4/16) should not affect the generality and the correctness of the model, because they reflect the different content of genome regions with slow, moderate, and fast replication rates. The additional restriction in the analyzed gene regions meets not only the economic expectations. The low number (5793) of finally classified records limits the number of classification attributes, which should be much smaller than the amount of data. We, therefore, decided to focus on the limited number of attributes related to the narrowed gene region responsible for the initiation of the transcription as a natural location of possible regulation of the expression rate.

The limited size of the analyzed data leaves the fundamental question of whether the attributes and routes found here by the method of machine learning, as very important, can sufficiently define the conditions for the accurate prediction of gene expression scores in a case of other organisms and experimental constraints. We believe that the first few parameters and pathways have already been determined, but many more remain to be explored for a broad range of organisms and environmental conditions.

## 5. Conclusions

In practice, a classification model for predicting gene expression scores in *S. cerevisiae* was developed on the basis of a set of variable physical, environmental, logistic, genetic, and statistical attributes, including transcripts, noncoding 5’ UTRs, coding nucleotides, and coding amino acid sequences; the properties and functions of synthesized proteins; and the experimental conditions. We expect that, in this way, we could also gain knowledge of the importance of the considered factors. We believe that the only source of knowledge is understanding the information obtained as a result of great experience; thus, the expected work should cover large datasets from large databases, deposited as needed resources. We decided to facilitate this study with the technique of machine learning (ML), the domain of AI being a natural area for processing big data.

The main conclusions from our work are as follows:

- The physical attributes of genes are the most important for determining gene expression scores when they are examined by different attribute evaluators.
- The random forest classification algorithm may be adapted to successfully predict the intensity of gene expression and indicate the best routes and parameters for this prediction.
- Logistic attributes dominate other attributes in random forest prediction, but the predictive role of attribute ensembles may be stronger than that of single attributes.
- The genetic type attributes may not be the most important in determining the expression score, but they cannot be ignored.
- The random forest classifier is a promising tool for in silico experiments.

## Figures and Tables

**Table 1:** Attribute selection (top ranking).

Mean Normalized Rank	Attribute	Meaning
0.02741	MM	the molecular mass of the protein
0.025221	ProtL	the length of the protein-coding sequence
0.017287	TransL	the length of the transcript
0.015501	Fun	the gene function
0.007004	nC	the number of the cytosine
0.006534	5’UTRL	the length of the 5’ untranslated region
0.02741	MM	the molecular mass of the protein
0.025221	ProtL	the length of the protein-coding sequence

**Table 2:** Final list of analyzed attributes.

Selected Aspects of Features and Conditions	Attribute	Meaning
Physical properties	TransL 5’UTRL ProtL MM	the length of the transcript the length of the 5’ untranslated region the length of the protein-coding sequence the molecular mass of the protein
Experimental conditions	Exp Time	the experiment type the time of the experiment
Logistic	Chro Fun	the order number of chromosome the gene function
Genetic	U3 U2 U1 AA2 S4 S5 S6	base of the 5’UTR (3rd before start codon) base of the 5’UTR (2nd before start codon) base of the 5’UTR (1st before start codon) amino acid (2nd coded by DNA, 1st after methionine) base of the coding sequence (4th, 1st after start codon) base of the coding sequence (5th, 2nd after start codon) base of the coding sequence (6th, 3rd after start codon)
Statistic	nA nT nG nC	the number of the adenine the number of the thymine the number of the guanine the number of the cytosine
Class	LMH	the low, moderate, and high expression scores (ES)

**Table 3:** Summary of classification attempts for the test set. Correctly classified instances, cci [%].

Classifier	cci [%]	Comments	References
ZeroR	33.3	Predicts the mean (for a numeric class) or the mode (for a nominal class). Constructs a frequency table for the target and select its most frequent value.	[29]
BayesNet	41.3	Uses various search algorithms and quality measures. Base class for a Bayes Network classifier. Provides data structures (network structure, conditional probability distributions, etc.) and facilities algorithms like K2 and B that are common to Bayes Network learning.	[30]
Logistic	28.6	Constructs and uses a multinomial logistic regression model with a ridge estimator.	[31]
MultilayerPerceptron	54.0	Uses backpropagation to classify instances. This network can be built by hand, created by an algorithm, or both. The network can also be monitored and modified during the training time. The nodes in this network are all sigmoid (except for when the class is numeric, in which case the output nodes become unthresholded linear units).	[30]
IBk	44.4	K-nearest neighbors’ classifier. Can select the appropriate value of K based on cross-validation. Can also conduct distance weighting.	[32]
Kstar	68.3	An instance-based classifier. The class of a test instance is based upon the class of those training instances similar to it, as determined by some similarity function. It differs from other instance-based learners in that it uses an entropy-based distance function.	[33]
OneR	58.7	Uses the minimum-error attribute for prediction, discretizing numeric attributes.	[34]
J48	65.1	Generates a pruned or unpruned C4.5 decision tree.	[35]
RandomForest	77.8	Constructs a forest of random trees.	[36]
RandomTree	42.9	Constructs a tree that considers K randomly chosen attributes at each node. Performs no pruning. Also has an option to allow the estimation of class probabilities (or the target mean in the regression case) based on a hold-out set (backfitting).	[30]

**Table 4:** The attribute importance is shown on the basis of the average impurity decrease and the number of nodes using that attribute for the optimal random forest model.

Importance	Number of Nodes	Attribute
0.73	6906	Exp
0.61	9276	Time
0.54	712	Fun
0.41	529	ProtL
0.41	717	TransL
0.4	411	MM
0.39	541	5’UTRL
0.39	160	U2
0.37	158	U3
0.37	189	AA2
0.35	150	Chro
0.35	146	S6
0.33	156	U1
0.32	109	nG
0.31	74	S4
0.3	50	S5
0.29	92	nC
0.29	163	nA
0.27	114	nT

**Table 5:** Detailed accuracy by class. Classified samples: true positive (TP), false positive (FP), true negative (TN), and false negative (FN) with the respective rates (R), and total accuracy (ACC).

Class	TP	FP	TN	FN	TPR	FPR	TNR	FNR	ACC
L	18	1	41	3	0.857143	0.02381	0.97619	0.142857	0.936508
M	21	9	33	0	1	0.214286	0.785714	0	0.857143
H	14	0	42	7	0.666667	0	1	0.333333	0.888889

**Table 6:** The best-predicted routes.

Class	Route	Attempts	False Predictions
L	ProtL < 2910 ProtL ≥ 514.5 nA ≥ 122.5 5’UTRL < 264.5	41	0
M	ProtL ≥ 2910 5’UTRL ≥ 64.5 Exp = Dia	120	0
H	S5 = T Exp = Dia U3 = A ProtL ≥ 469.5 MM < 71,288.1 5’UtrL ≥ 72	71	0

## Data Availability

All data generated or analyzed during this study are included in this article. Further inquiries can be directed to the corresponding author.

## References

[1] Crick F. (1970). Central Dogma of Molecular Biology. Nature.

[2] Ralston A., Shaw K. (2008). Gene expression regulates cell differentiation. Nat. Educ..

[3] Wright J. (2020). Gene Control.

[4] Phillips T. (2008). Regulation of transcription and gene expression in eukaryotes. Nat. Educ..

[5] Paudel B.P., Xu Z.-Q., Jergic S., Oakley A.J., Sharma N., Brown S.H.J., Bouwer J.C., Lewis P.J., Dixon N.E., van Oijen A.M. (2022). Mechanism of transcription modulation by the transcription-repair coupling factor. Nucleic Acids Res..

[6] Whiteside S.T., Goodbourn S. (1993). Signal transduction and nuclear targeting: Regulation of transcription factor activity by subcellular localisation. J. Cell Sci..

[7] Kim S., Kaang B.K. (2017). Epigenetic regulation and chromatin remodeling in learning and memory. Exp. Mol. Med..

[8] Bumgarner R. (2013). Overview of DNA microarrays: Types, applications, and their future. Curr. Protoc. Mol. Biol..

[9] Wang Z., Gerstein M., Snyder M. (2009). RNA-Seq: A revolutionary tool for transcriptomics. Nat. Rev. Genet..

[10] (2025). SGD Projekt. https://sites.google.com/view/yeastgenome-help/function-help/expression-data.

[11] Siwiak M., Zielenkiewicz P. (2010). A Comprehensive, Quantitative, and Genome-Wide Model of Translation. PLoS Comput. Biol..

[12] Minca E.C., Al-Rohil R.N., Wang M., Harms P.W., Ko J.S., Collie A.M., Kovalyshyn I., Prieto V.G., Tetzlaff M.T., Billings S.D. (2016). Comparison between melanoma gene expression score and fluorescence in situ hybridization for the classification of melanocytic lesions. Mod. Pathol..

[13] Siwiak M., Zielenkiewicz P. (2013). Transimulation-Protein Biosynthesis Web Service. PLoS ONE.

[14] Mehdi A.M., Patrick R., Bailey T.L., Bodén M. (2014). Predicting the Dynamics of Protein Abundance Technological Innovation and Resources. Mol. Cell. Proteom..

[15] Li W., Yin Y., Quan X., Zhang H. (2019). Gene Expression Value Prediction Based on XGBoost Algorithm. Front. Genet..

[16] Mitchell T. (1997). Machine Learning.

[17] Chicco D. (2017). Ten quick tips for machine learning in computational biology. BioData Min..

[18] Ho T.K. Random Decision Forests (PDF). Proceedings of the 3rd International Conference on Document Analysis and Recognition.

[19] Spiesser T.W., Diener C., Barberis M., Klipp E. (2010). What Influences DNA Replication Rate in Budding Yeast?. PLoS ONE.

[20] Laso M.V., Zhu D.E.L.I.N., Sagliocco F., Brown A.J., Tuite M.F., McCarthy J.E. (1993). Inhibition of translational initiation in the yeast Saccharomyces cerevisiae as a function of the stability and position of hairpin structures in the mRNA leader. J. Biol. Chem..

[21] Johnson A., Lewis J., Alberts B. (2002). The Shape and Structure of Proteins. Molecular Biology of the Cell.

[22] Eibe F., Hall M.A., Witten I.H. (2016). The WEKA Workbench. Online Appendix for “Data Mining: Practical Machine Learning Tools and Techniques”.

[23] Oromendia A.B., Dodgson S.E., Amon A. (2012). Aneuploidy causes proteotoxic stress in yeast. Genes Dev..

[24] Kunkel J., Luo X., Capaldi A.P. (2019). Integrated TORC1 and PKA signaling control the temporal activation of glucose-induced gene expression in yeast. Nat. Commun..

[25] Gasch A.P., Spellman P.T., Kao C.M., Carmel-Harel O., Eisen M.B., Storz G., Botstein D., Brown P.O. (2000). Genomic expression programs in the response of yeast cells to environmental changes. Mol. Biol. Cell.

[26] Cherry J.M., Adler C., Ball C., Chervitz S.A., Dwight S.S., Hester E.T., Jia Y., Juvik G., Roe T., Schroeder M. (1998). SGD: Saccharomyces Genome Database. Nucleic Acids Res..

[27] Yanofsky C. (2007). Establishing the Triplet Nature of the Genetic Code. Cell.

[28] Edgar R., Domrachev M., Lash A.E. (2002). Gene Expression Omnibus: NCBI gene expression and hybridization array data repository. Nucleic Acids Res..

[29] Nasa C., Suman (2012). Evaluation of Different Classification Techniques for WEB Data. Int. J. Comput. Appl..

[30] (2025). WEKA. https://weka.sourceforge.io/doc.dev/index.html?overview-summary.html.

[31] le Cessie S., van Houwelingen J.C. (1992). Ridge Estimators in Logistic Regression. Appl. Stat..

[32] Aha D., Kibler D. (1991). Instance-based learning algorithms. Mach. Learn..

[33] Cleary J.G., Leonard E. Trigg: K*: An Instance-based Learner Using an Entropic Distance Measure. Proceedings of the 12th International Conference on Machine Learning.

[34] Holte R.C. (1993). Very simple classification rules perform well on most commonly used datasets. Mach. Learn..

[35] Quinlan R. (1993). C4.5: Programs for Machine Learning.

[36] Breiman L. (2001). Random Forests. Mach. Learn..

[37] Mantione K.J., Kream R.M., Kuzelova H., Ptacek R., Raboch J., Samuel J.M., Stefano G.B. (2014). Comparing bioinformatic gene expression profiling methods: Microarray and RNA-Seq. Med. Sci. Monit. Basic. Res..

[38] Bergemann T.L., Wilson J. (2011). Proportion statistics to detect differentially expressed genes: A comparison with log-ratio statistics. BMC Bioinform..

[39] Koch C.M., Chiu S.F., Akbarpour M., Bharat A., Ridge K.M., Bartom E.T., Winter D.R. (2018). A Beginner’s Guide to Analysis of RNA Se-quencing Data. Am. J. Respir. Cell Mol. Biol..

[40] Quackenbush J. (2002). Microarray data normalization and transformation. Nat. Genet..

[41] Li J., Liang Q., Song W., Marchisio M.A. (2017). Nucleotides upstream of the Kozak sequence strongly influence gene expression in the yeast S. cerevisiae. J. Biol. Eng..

[42] Arhondakis S., Auletta F., Torelli G., D’Onofrio G. (2004). Base composition and expression level of human genes. Gene.

[43] Singh R., Sophiarani Y. (2020). A report on DNA sequence determinants in gene expression. Bioinformation.

